# Criteria for the Classification of the Interradicular Septum Shape in Maxillary Molars with Clinical Importance for Prosthetic-Driven Immediate Implant Placement

**DOI:** 10.3390/diagnostics12061432

**Published:** 2022-06-10

**Authors:** Jovana Milenkovic, Milica Vasiljevic, Nemanja Jovicic, Dragan Milovanovic, Dragica Selakovic, Gvozden Rosic

**Affiliations:** 1 Department of Dentistry, Faculty of Medical Sciences, University of Kragujevac, 34000 Kragujevac, Serbia; jov.mil.2022@gmail.com (J.M.); milicavaska13@gmail.com (M.V.); 2 Department of Histology and Embryology, Faculty of Medical Sciences, University of Kragujevac, 34000 Kragujevac, Serbia; nemanjajovicic.kg@gmail.com; 3 Clinical Pharmacology Department, Clinical Centre Kragujevac, 34000 Kragujevac, Serbia; piki@medf.kg.ac.rs; 4 Department of Pharmacology and Toxicology, Faculty of Medical Sciences, University of Kragujevac, 34000 Kragujevac, Serbia; 5 Department of Physiology, Faculty of Medical Sciences, University of Kragujevac, 34000 Kragujevac, Serbia

**Keywords:** interradicular septum, prosthetic-driven implant therapy, CBCT

## Abstract

The aim of this study was to use cone-beam computed tomography (CBCT) to evaluate the morphometric properties of the interradicular septum (IRS) in the maxillary molar region that may be indicative for prosthetic-driven implant placement. Following the repetitive algorithm based on the visual identification of IRS shapes, we described the following IRS shapes: arrow, boat, drop, and palatal and buccal convergence. The incidence of IRS shapes showed significant differences for the first and second maxillary molars (the highest frequency for the arrow shape, and the lowest for the drop shape) with no significant difference between the molars. The most prominent width indicative for implant placement was observed in the palatal convergence shape, whereas the height criteria were the most satisfying in the buccal convergence-shaped IRS for both molars. Apart from the parameters in the coronal view, the image analysis in the axial view revealed that IRS surface area, required for the implant placement, was the most prominent in the palatal convergence shape for the first, and boat shape for the second molars. Our results showed the benefits of CBCT diagnostics in posterior maxilla morphometric analysis. IRS shape classification may be helpful in achieving more rapid and accurate planning for interventions in this region.

## 1. Introduction

Maxillary molars present wide anatomical variability, especially when it comes to heterogeneity in the furcation topography [1]. Since the upper molars are multi-rooted teeth, predominantly with three roots (mesial, distal, and palatal), their anatomy is obviously very complex [2]. From the aspect of implantology in this specific region, numerous potential implant sites were reported in the post-extractive alveoli structure [3,4]. However, according to the literature data, the interradicular septum (IRS) is usually considered to be the best fixture position from the prosthetic point of view [5,6,7,8]. As the IRS of the maxillary molars expresses significant morphometric variability (height and width [9]), preoperative radiological analyses are highly recommended [10]. In the last decades, cone-beam computed tomography (CBCT) has offered a possibility to obtain a more detailed radiological evaluation of anatomical structures, especially in the oral and maxillofacial area, when compared to conventional 2D radiography [11,12]. Apart from the significantly higher accuracy and reproducibility, the advantages of CBCT also include lower radiation doses, easier image acquisition, and multiplanar reconstruction over computed tomography, as well as lower costs [13,14,15,16]. Rajkovic Pavlovic and collaborators [9] recently reported that CBCT provides a possibility to achieve detailed IRS morphological characteristics in the region of the posterior maxilla, which may significantly impact the characteristics of the implant.

As stated above, the IRS is considered to be the ideal site for prosthetic-driven implant placement in the maxillary molar region [17]. Therefore, it is not surprising that numerous studies estimated IRS architectures [7,18]. Namely, Amato and coworkers [19] noticed that over 60% of molars had an IRS height of less than 7 mm. On the other hand, Rajkovic Pavlovic and colleagues [20] highlighted the importance of the implant length required to achieve primary implant stability. Since the recommended minimal implant length for prime implant fixation is 10 mm [21], it can be implied that immediate implant engagement in the IRS may often require the elevation of the sinus floor [22]. Those combined therapeutic procedures, under the described circumstances, may have considerable importance for safe implant placement as well as for the reduction of sinus membrane perforation risks [23]. IRS width, another IRS property that can be easily obtained by CBCT analysis, also plays an important role in the prognosis of the implant therapy’s success, as Hayacibara and coworkers [24] reported an IRS width of 3 mm as the minimum limit that can provide initial implant stability. Since in most cases the IRS width in the region of maxillary molars is less than 3 mm (especially for the second molars [20]), this structure may usually require additional surgical procedures, such as osseodensification [25], to provide adequate implant stability.

The current literature offers several classifications according to the IRS dimensions. Smith and Tarnow [26] described three categories of molar extraction sockets, based on the quantity of IRS available for typical implant placement (but with no quantification of IRS characteristics). Type A sockets have sufficient septal bone bulk for the total implant surface placement. Type B sockets also allow enough septal bone bulk to achieve implant stability, but do not cover the total implant surface (with possible appearance of gaps), whereas type C sockets have insufficient septal bone space as is required to stabilize the implant without engaging the socket walls. On the other hand, Bleyan and colleagues [25] proposed a new IRS classification, based exclusively on IRS width, and reported four categories as follows: S-I—septum initial width above 4 mm; S-II—septum initial width 3–4 mm; S-III—septum initial width 2–3 mm; and S-IV—septum initial width below 2 mm or no septal bone present.

Obviously, the existing classifications are based on clinical evaluations of the post-extractive alveoli structure. However, there is a need for preclinical investigations that can provide an accurate determination of IRS morphometric characteristics prior to any intervention that may allow more precise and reliable planning of the prosthetic-driven immediate implant placement procedures. Thus, the aim of this study was to evaluate, by using CBCT, the morphometric properties of the IRS in the region of maxillary molars, which may have clinical importance with regards to the prosthetic-driven implant therapy aspect. 

## 2. Materials and Methods

### 2.1. The Ethical Committee

The research was approved by the Ethical Committee of the Faculty of Medical Sciences, the University of Kragujevac, Serbia (ID:01-3568).

### 2.2. The Sample Characteristics

This was a retrospective study, which obtained CBCT scans from a radiological database of the Department of Dentistry, University of Kragujevac, Serbia in March and April 2022. The patients came to the radiological evaluation for different reasons including: planning implant therapy, oral and maxillofacial surgery, and endodontic, prosthodontic, and orthodontic interventions. The inclusion criteria were: subjects over 18 years old and the presence of the first and/or second maxillary molars. On the other hand, the presence of dental and maxillofacial pathology in the area of maxillary molars (tooth destruction, interradicular resorption, periapical lesion, cyst, tumor, trauma, etc.), bone grafts, bone surgical interventions, dental implants in the area of maxillary molars, patients with systemic disease (osteoporosis), and low image quality of CBCT scans were considered as exclusion criteria. Following these criteria, the study included 173 patients (88 male and 85 female, with average ages of 44.95 ± 1.70 and 41.59 ± 1.44 years, respectively) with 353 first and second maxillary molars (160 and 193, respectively).

### 2.3. The CBCT Device and Software Characteristics

All CBCT scans were obtained using an Orthophos XG 3D device (Sirona Dental Systems GmbH, Bensheim, Germany), with 3D settings for recording, either VOL1 HD (85 kV/6 mA, exposure time—14.3 s.) or VOL2 HD (85 kV/10 mA, exposure time—5.0 s.), and a voxel size of 160 µm or 100 µm, respectively. The field of view for all CBCT scans was 8 × 8 cm. CBCT images were analyzed using GALAXIS software v1.9.4 (Sirona Dental Systems GmbH, Bensheim, Germany).

### 2.4. The Morphometric Characteristics of the Maxillary Molars Interradicular Septum

**Figure 1:**
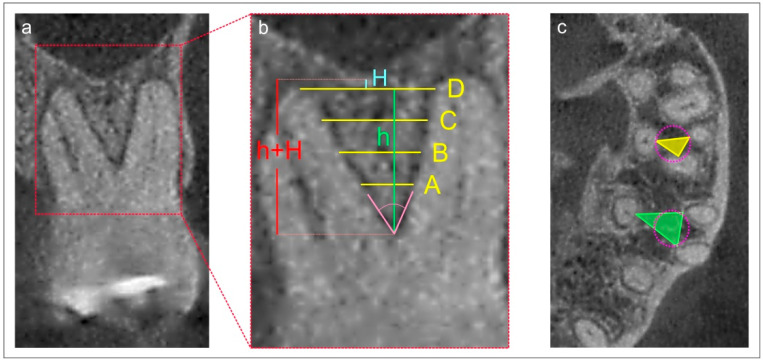
The morphometric evaluation of CBCT images of the maxillary molar region in the coronal view (CBCT image—**a**, with landmarks—**b**), and in the axial view (**c**). The morphometric evaluation of CBCT images in the axial view, at the C level (the triangle represents the calculated IRS surface area—green for the first molars; yellow for the second; the commonly used implant diameter in the posterior maxilla is encircled).

The maxillary molars’ interradicular septum morphometric characteristics were evaluated according to the previously described methodology [8,20]. Namely, in the coronal plane, we performed linear measurements (in mm, except for the angle in °) from the CBCT images as follows (Figure 1a,b):

1. IRS width at the A level (2 mm from the interradicular furcation);
2. IRS width at the B level (midpoint of IRS height);
3. IRS width at the C level (2 mm from the IRS base);
4. IRS width at the D level (IRS base);
5. IRS height—h (the distance between the interradicular furcation and IRS base);
6. The distance between IRS base and sinus floor—H;
7. Interradicular furcation angle.

In addition, we calculated the distance between the interradicular furcation and the sinus floor (H + h, in mm).

Furthermore, we evaluated IRS morphometric characteristics obtained in the axial plane, and after radiological reconstruction we used Heron’s formula [27,28,29] to estimate IRS surface area (Figure 1c). As shown in Figure 1c, we compared the commonly used implant diameter in the posterior maxilla [30,31] (4 mm diameter = 12.56 mm^3^ surface area) and IRS surface area to present the clinical issue for the prosthetic-driven immediate implant placement concept. All parameters were analyzed by two independent observers blind to the protocol, with high inter-rater reliability (Pearson’s r = 0.95).

As our visual impressions sufficiently indicated that there was significant regularity in IRS shape according to the evaluated parameters, we also tried to make the classification of IRS shapes based on clinical importance according to the literature data.

### 2.5. Statistical Analysis

The data presented herein were expressed as the means ± SEM. The parameters were initially submitted to Levene’s test for homogeneity of variance and to the Shapiro–Wilk test of normality. The comparisons between the groups were performed using the chi-square test or one-way ANOVA, followed by Scheffe’s post hoc test. Furthermore, Pearson’s coefficient of correlation was used to analyze relationships between parameters, and simple linear regression analyses were performed. A *p* value < 0.05 was considered significant. Statistical analysis was performed with the SPSS version 20.0 statistical package (IBM SPSS Statistics 20, Armonk, NY, USA).

## 3. Results

The initial insight into the parameters obtained in the area of interest (maxillary molars’ IRS, as previously described by Rajkovic Pavlovic and coworkers [20]) led us to a certain and repetitive algorithm that we tried to evaluate in general prior to further analyses. Thus, in order to achieve the quantitative criteria that would allow maxillary molars’ IRS shape classification (Table 1) and in order to provide the analysis of individual parameter alterations depending on the IRS shape, we proposed the following numeric determinants for the visually identified shapes (i.e., arrow, boat, drop, and palatal and buccal convergence), as shown in Table 1.

**Figure 2:**
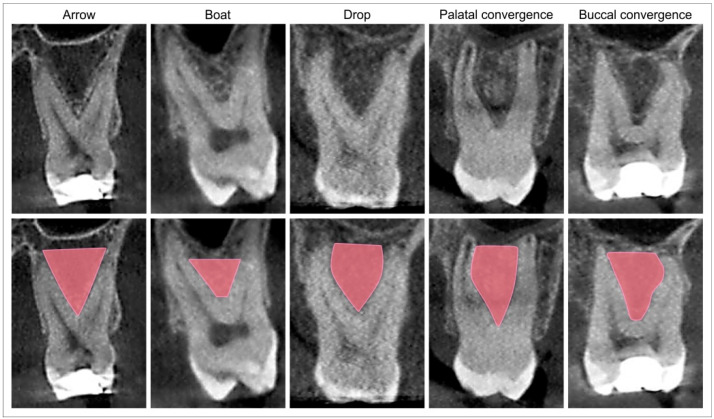
Typical maxillary molars’ IRS shapes (with the marked borders) in the coronal CBCT view.

The representative images for each proposed IRS shape are shown in Figure 2.

Interestingly, the incidence of various maxillary molars’ IRS shapes showed significant differences for both the first (chi-square = 72.5, df = 4, *p* < 0.001) and the second (chi-square = 66.8, df = 4, *p* < 0.001) maxillary molars (Table 2), with the arrow shape having the notably highest frequency and the drop shape having the lowest frequency, and there was no significant difference in the incidence between the M1 and M2 for each IRS shape (chi-square = 1.3, df = 4, *p* = 0.855).

**Figure 3:**
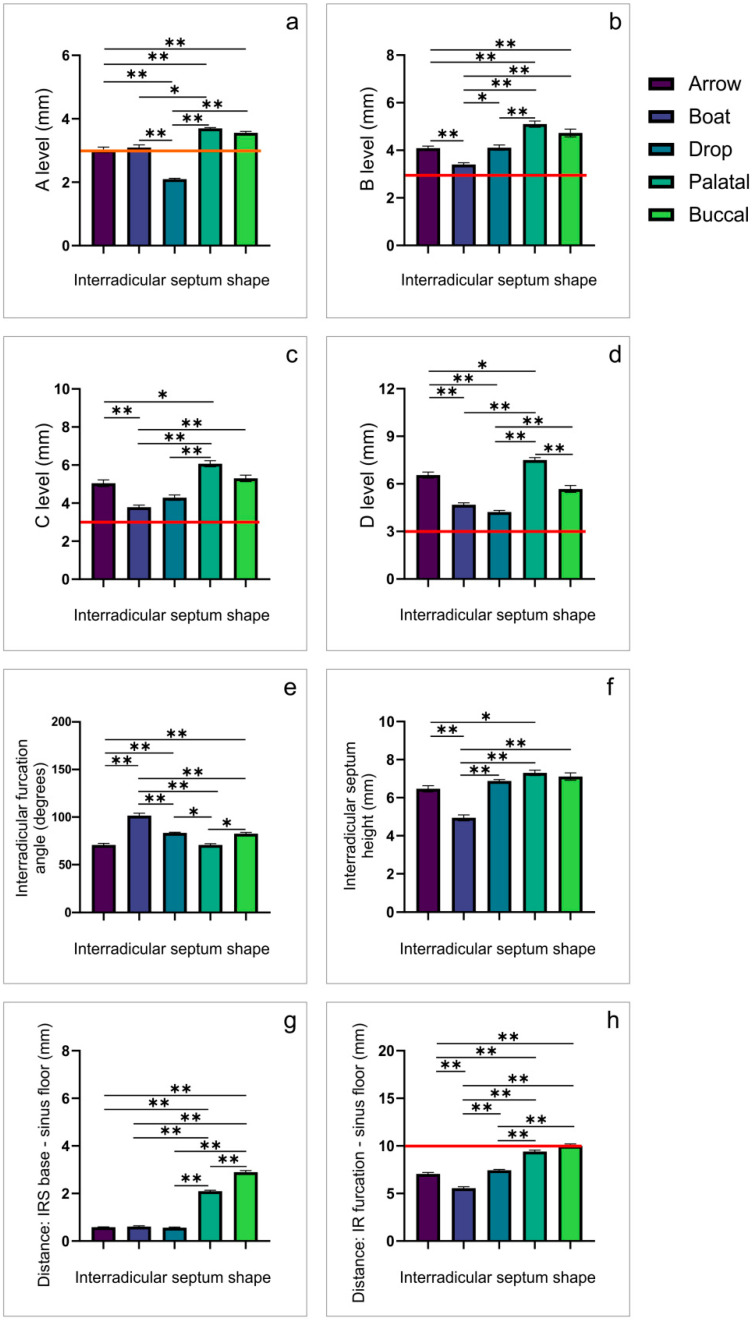
The observed differences in IRS morphometric parameters according to the IRS shape for the first maxillary molars obtained in the coronal CBCT views (the margin of clinical importance). (**a–d**)—IRS width at levels A, B, C, and D, respectively; (**e**)—IR furcation angle; (**f**)—IRS height; (**g**)—the distance between IRS base and the sinus floor; (**h**)—the distance between IR furcation and the sinus floor. * Denotes a significant difference of *p* < 0.05, ** denotes a significant difference of *p* < 0.01.

Following the described methodology for the maxillary molars’ IRS analysis [20], the estimation of coronal views for the first maxillary molars revealed the fact that IRS shape significantly affected the dimensions of IRS at all estimated levels (A, B, C, and D; df = 4, F = 27.744, 22.078, 12.740, and 28.041, respectively). As shown in Figure 3, the diameter determined at level A was significantly lower in the drop when compared to other IRSs. On the other hand, the boat-shaped diameter was accompanied with the lowest values at levels B and C when compared to the other IRS shapes. Again, both boat and drop shapes expressed values significantly below the other IRS shapes at the D level. In contrast, the analysis of the IR furcation angle revealed that the boat IRS shape was significantly above the values observed in all other estimated shapes. At the same time, the values observed for IRS height and the distance between the IRS base and the sinus floor, as well as the total distance between the IR furcation and the sinus floor in the boat-shaped IRS was significantly below the other IRS shapes. In contrast, both palatal and buccal convergence shapes expressed the highest total distance between the IR furcation and the sinus floor when compared to other shapes, predominantly due to significantly higher values for the distance between the IR septum base and the sinus floor.

**Figure 4:**
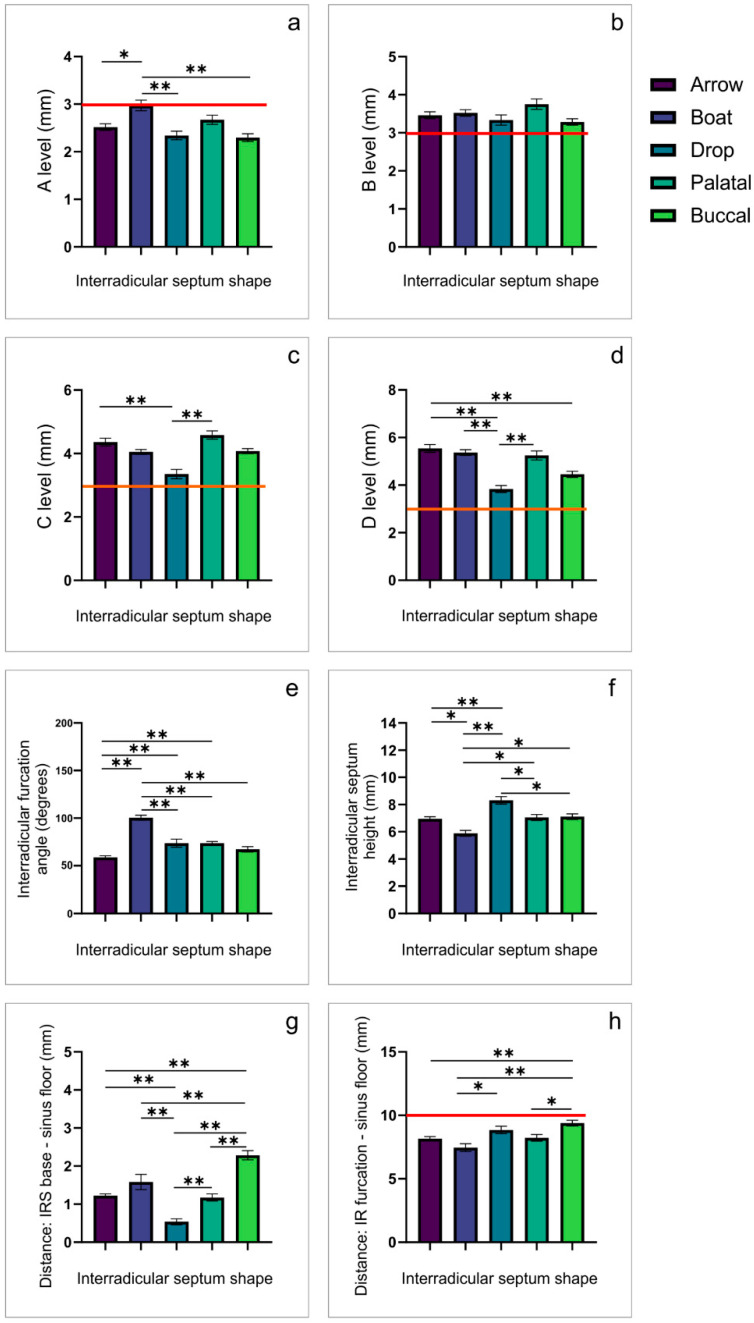
The observed differences in IRS morphometric parameters according to the IRS shape for the second maxillary molars obtained in the coronal CBCT view (the margin of clinical importance). (**a**–**d**)—IRS width at levels A, B, C, and D, respectively; (**e**)—IR furcation angle; (**f**)—IRS height; (**g**)—the distance between IRS base and the sinus floor; (**h**)—the distance between IR furcation and the sinus floor. * Denotes a significant difference of *p* < 0.05, ** denotes a significant difference of *p* < 0.01.

The estimation of IRS images for the M2 in the coronal view (Figure 4) was also significantly affected by IRS shape at all estimated IRS levels (A, B, C and D; df = 4, F = 5.883, 2.083, 7.894, and 12.276, respectively). The analysis revealed that the drop IRS shape was accompanied by the significantly lower values for the IRS diameter when compared to other IRS shapes at estimated levels (except for level B). In contrast, the boat-shaped IRS showed a significantly wider IR furcation angle than other IRS shapes. The analysis of M2 vertical diameters obtained in the coronal view resulted in a quite complex outcome. Thus, the drop IRS shape was accompanied with the highest values for IRS height and, at the same time, the lowest values for the distance between the IR septum base and the sinus floor, resulting in the highest mean values (along with the buccal convergence shape) for the total distance between the IR furcation and the sinus floor when compared to other IRS shapes.

**Figure 5:**
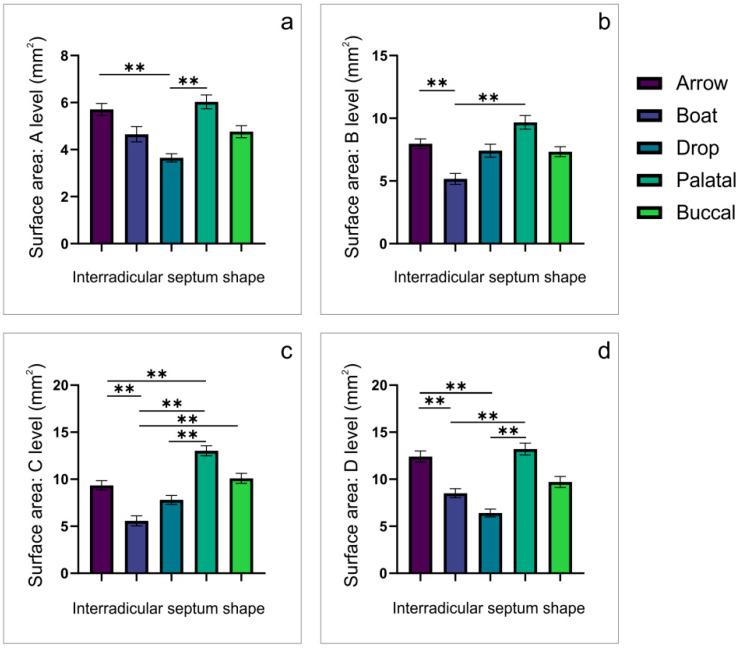
The observed differences in IRS surface area for the first maxillary molars obtained in the axial CBCT views. (**a**)—at the A level; (**b**)—at the B level; (**c**)—at the C level; (**d**)—at the D level. ** denotes a significant difference of *p* < 0.01.

Not surprisingly, the analysis of the first maxillary molars’ IRS images (Figure 5) in the axial view confirmed that the IRS shape significantly influenced the IRS surface area of all estimated levels (A, B, C and D; df = 4, F = 7.167, 7.502, 14.319, and 12.973, respectively). Namely, two specific IRS shapes, the boat and the drop, were accompanied with the lowest values of the surface areas, which were significantly below the other IRS shapes. However, although the drop-shaped IRS achieved the lowest surface area at levels A and B, the lowest values for surface area in the boat-shaped IRS was achieved at levels B and C.

**Figure 6:**
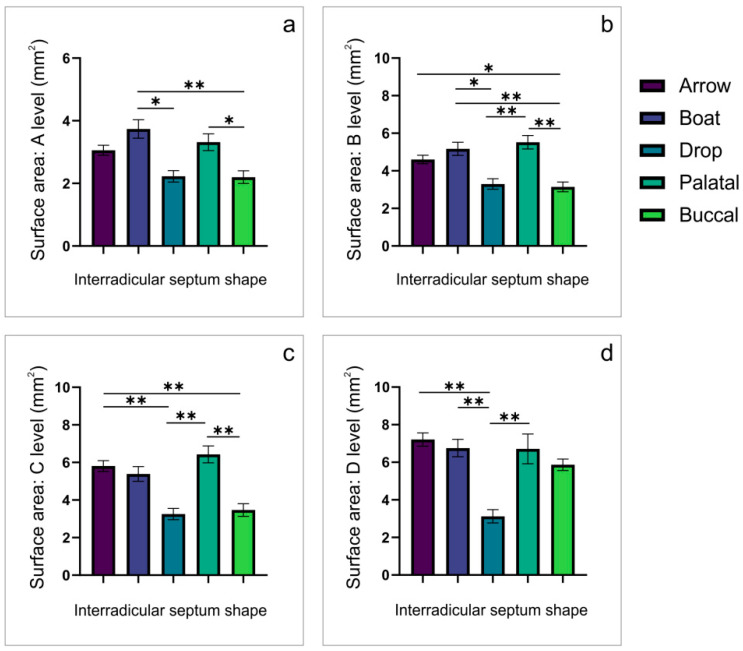
The observed differences in IRS surface area for the second maxillary molars obtained in the axial CBCT views. (**a**)—at the A level; (**b**)—at the B level; (**c**)—at the C level; (**d**)—at the D level. * Denotes a significant difference of *p* < 0.05, ** denotes a significant difference of *p* < 0.01.

Unlike for the M1, a significant impact of IRS shape on the surface areas observed at all estimated M2 IRS levels (A, B, C, and D; df = 4, F = 6.239, 9.295, 11.501, and 7.620, respectively) followed a simpler algorithm (Figure 6). Thus, the lowest values for the surface areas appeared in the drop-shaped IRS at all estimated levels. Except for at the D level, this regularity was also followed in the buccal convergence IRS shape.

Following the previously established criteria that emphasized numeric borders for the prognosis of the immediate implant placement success, which had set the minimal value for IRS width at 3 mm [32] and the vertical axis value at 10 mm [21], we presented the analysis of our results on the basis of IRS shape influence on those determinants obtained in the coronal views. The analysis of the average values of the appropriate parameters for M1 (Table 3) showed that critical criteria were not supposed to be achieved in the drop-shaped IRS at level A, whereas the risks for the immediate implant placement complications, according to the total distance between the IR furcation and the sinus floor, were the lowest in the buccal-convergence-shaped IRS.

As expected, the analysis following the same criteria for the prediction of the immediate implant placement success at the M2 position revealed insufficient values for IRS diameter only at level A (except for the boat-shaped IRS), as well as for the critical vertical diameter (Table 4).

For the same purposes, we also performed the analysis of the metrics obtained in the axial view for both M1 (Table 5) and M2 (Table 6). Again, following the previously established criteria for the minimal required IRS surface for the immediate implant placement [32], it could be noted that the critical values for the surface area should be expected at all IRS levels (for all IRS shapes), except for the palatal convergence shape on the C and D level (Table 5). However, it should be noted that the most insufficient space, when compared to the implant surface, appeared at level A, especially for the drop-shaped IRS. Not surprisingly, following average values for the surface area obtained for the M2 IRS (Table 6), a similar regularity was observed also for M2 IRS. Likewise, the most critical IRS surface area values should be expected at levels A and B.

## 4. Discussion

Significant improvement in various fields of dentistry has been achieved by employing new diagnostic procedures, including CBCT which has already become a standard methodology. To obtain more detailed morphological and morphometric information about the IRS in the region of the posterior maxilla, we used CBCT images for an advanced analysis. The current literature data has shown the validity of CBCT methodology in the evaluation of the shape of anatomical structures in the maxillofacial region [16,33,34,35]. Namely, in the anterior maxilla, Mardinger and collaborators [36] established NPC shapes in the sagittal CBCT view, whereas Von Arx and coworkers [37] defined the shapes of accessory canals in the coronal CBCT view. The main methodology used in this study was based on the previous investigation by Regnstrand and colleagues [38] who performed the analysis of the maxilla posterior parts (including maxillary sinus) using CBCT image analyses at the three orthogonal planes. However, even before the presentation of the maxillary molars’ IRS morphometric parameters, certain regularity was noticed for both the first and the second maxillary molars’ IRS when considering their values according to how visually easy it was to identify shapes of IRS structures. Therefore, we tried to apply systematic criteria to the IRS shape in the region of maxillary molars, which may be accompanied by the alterations in IRS diameters, due to the potential clinical importance in the prosthetic-driven implant therapy. To our knowledge, this is the first study that described the impact of different categories of maxillary molars’ IRS shape on the parameters that determine the implant placement planning.

According to the numeric determinants (the diameter of IRS at the D level and the IR furcation angle, Table 1) for the visually identified shapes (Figure 2), we proposed the classification of IRS shapes into five categories: arrow, boat, drop, palatal convergence, and buccal convergence. Additional analysis showed that every IRS shape was accompanied with the specific morphometric characteristics obtained in both coronal (Figure 3 for the first maxillary molars and Figure 4 for the second maxillary molars) and axial (Figure 5 for the first maxillary molars and Figure 6 for the second maxillary molars) views. Accordingly, in the arrow-, boat-, palatal-convergence-, and buccal-convergence-shaped IRS, the diameter at the D level was above 4 mm, whereas only the drop-shaped IRS width at the D level was bellow 4 mm in both the first and the second maxillary molars. Interestingly, the IRS furcation angle showed more variations according to IRS shape in the first maxillary molars (60–90°) when compared to the second maxillary molars (critical value established at 70°).

The IRS shape distribution in the first maxillary molars, as well as in the region of the second molars, presented significant differences. Namely, the arrow-shaped IRS was predominant, whereas the drop shape was the least frequent in both the first and the second molars. Since there was no data from the literature to compare either numerically or generically with our results, we could only speculate that the reason for this observation could be found in the similarity of the morphological root characteristics in the first and second maxillary molars [39], due to fact that the IRS furcation angle strongly depends on the root shape.

In order to provide a better insight into IRS shape classification by means of its clinical applicability, we performed consecutive horizontal linear measurements in the coronal plane. We used the critical IRS width for initial implant stability of 3 mm as the reference point, as previously described [32]. When applying this criterion for the first maxillary molars at the A level (Figure 3, the red horizontal line presents the border of 3 mm), it could be noticed that the drop-shaped IRS in the first maxillary molars was accompanied with the insufficient IRS width to achieve primary stability during immediate implant placement, whereas the arrow- and boat-shaped IRS presented a width approximately 3 mm (within the critical margins). Following Bleyan and coworkers’ IRS width classification established following extraction [25], it was found that arrow-, boat-, and drop-shaped IRS (at the A level) belong to the S-III septum category (initial width 2–3 mm). On the other hand, for the second maxillary molars, all estimated IRS shapes at the A level belong to the S-III septum category [25]. According to those findings, identification of IRS shape may be a reliable checkpoint for planning the interventions that require interradicular septum expansion using the osseodensification technique in order to allow successful immediate implant placement into the interradicular septum.

Besides the horizontal IRS diameters (where the lowest, the A level, is by far the most critical), another clinical criterion that could be analyzed in the coronal view is based on the IRS vertical characteristics. However, we presented two individual parameters that determine vertical axis (the distance between the interradicular furcation and the sinus floor) as the clinical importance relies on their sum, which represents the IRS thickness between the IRS furcation angle and the sinus floor, since it should meet the implant length. Nunes and coworkers [21] reported that the minimum bone height required to achieve primary stability and resistance to occlusal force is 10 mm. According to our results, it is evident that only the buccal convergence IRS shape had a 10 mm height for the first molars (Figure 3), whereas none of the IRS shapes expressed sufficient height in the second molars (Figure 4). The clinical importance of the presented data implies that prosthetic-driven immediate implant placement in the region of the second molars (Table 4) may require necessary additional surgical interventions, such as sinus floor elevation, whereas this supportive treatment may not be necessary for all IRS shapes in the first molars (Table 3). Furthermore, the results obtained in this study, considering the vertical diameter of the IRS according to its shape, allow the prediction of the extent of the contextual intervention that is necessary to achieve the implant stability (Table 3 and Table 4).

Using the axial CBCT slices, we evaluated IRS surface area according to the IRS shapes. For the first upper molars, the most prominent surface area at all estimated levels was observed for the palatal convergence IRS shape. In contrast, the drop (at the A and D levels) and the boat IRS shape (at the B and C levels) expressed the smallest surface area. On the other hand, the surface area in the second upper molars showed more diversity according to IRS shape. At the A level, the largest IRS surface area was observed in the boat IRS shape, and at the B and C levels for the palatal convergence. At the D level the largest surface area appeared in the arrow-shaped IRS. In contrast, the smallest surface area at all estimated levels was noticed in the drop-shaped IRS. The observed differences in the surface area according to IRS shape may have significant clinical importance, since the horizontal diameters of IRS may be crucially affected by implant width during the prosthetic-driven immediate implant placement. As the most frequent implant diameter in the region of the maxillary molars is 4 mm [30,31], we used a formula to calculate implant surface area [40] and to compare it with the IRS surface area. For the first maxillary molars (Table 5), only palatal convergence-shaped IRS allowed sufficient surface area achieved at the higher (C and D) levels, whereas the second maxillary molars expressed a smaller horizontal diameter than required for implants of standard dimensions (Table 6).

Although the IRS represents an ideal implant site for prosthetic aspects [41], our results indicate that the precise morphometric analysis of the maxillary molars’ IRS may allow the prediction of the prosthetic-driven immediate implant placement’s outcome. One of the checkpoints in the planning of the therapeutic approach may be based on the preliminary distinction of IRS shapes, since it may be decisive for both horizontal and vertical IRS characteristics in the region of maxillary molars and which may affect the implant stability. Due to the complexity of this therapeutic approach, it is not surprising that some authors [42,43] suggested other implant sites to avoid the loss of the IRS while drilling, but this approach does not fulfill the criteria for the prosthetic-driven concept of immediate implant placement. In order to preserve the prosthetic-driven protocol (and IRS as the implant site), different treatment options have been proposed. Thus, Fugazzotto and colleagues [44] proposed the insertion of the appropriate bur (with adequate angle) in the IRS, whereas Sanz and collaborators [45] reported that the space between the implant and IRS could be closed with bone grafting procedures. In line with the efforts to achieve the ideal implant site for prosthetic aspects using prosthetic-driven immediate implant placement, we presented the classification of IRS shapes in the maxillary molar region. This classification implies that certain IRS shapes are accompanied by characteristic horizontal and vertical diameters that may allow better planning of implant placement procedures. Additional information that will allow for a more reliable prognosis of the implant placement’s outcome, according to IRS shape, could be achieved by applying the methods of virtual implant placement in the region of the posterior maxilla.

## 5. Conclusions

According to the results of this study, the benefits of CBCT diagnostic in morphometric analysis of the posterior maxilla, accompanied by the proposed IRS shape classification, may be helpful in achieving more rapid and accurate planning for the interventions in this region. Even more, CBCT image analysis can provide significant therapeutic potential related to prosthetic-driven surgical interventions. The morphometric characteristics of maxillary molars IRS markedly depend on IRS shape, which in turn may be a substantially important checkpoint for the prosthetic-driven immediate implant placement. Still, further investigations performed on more extensive sample size (including ethnicity variations) should bring us to more reliable data with more accurate clinical relevance.

## Figures and Tables

**Table 1:** The quantitative criteria for maxillary molars’ IRS shape classification obtained in the coronal (D level and IR furcation angle) CBCT view.

The Interradicular Septum Shape	D Level		Angle	
	M1	M2	M1	M2
Arrow	≥4 mm	≥4 mm	≤60°	≤70°
Boat	≥4 mm	≥4 mm	≥90°	≥70°
Drop	≤4 mm	≤4 mm	≥70°	≤70°
Palatal convergence	≥4 mm	≥4 mm	≤70°	≤70°
Buccal convergence	≥4 mm	≥4 mm	≥60°	≤70°

**Table 2:** The incidence of maxillary molars’ IRS shapes in the first (M1) and the second (M2) maxillary molars.

IRS Shape	Arrow	Boat	Drop	Palatal Convergence	Buccal Convergence
**M1 (%)**	46.9	13.1	11.9	14.4	13.8
**M2 (%)**	43	12.4	11.4	18.7	14.5

**Table 3:** The relationship between the average values of the IRS parameters obtained in the coronal view and the minimal recommended IRS diameters for the first maxillary molars according to the IRS shape (critical points are colored). The last column represents the value of vertical diameter required to achieve implant stability (above 10 mm).

The Interradicular Septum Shape	Level A	Level B	Level C	Level D	10 − (h + H)
Arrow	3 mm	4 mm	5 mm	6.5 mm	3 mm
Boat	3 mm	3.4 mm	3.8 mm	4.7 mm	4.5 mm
Drop	2 mm	4 mm	4.3 mm	4.2 mm	2.5 mm
Palatal convergence	3.7 mm	5 mm	6 mm	7.5 mm	0.5 mm
Buccal convergence	3.5 mm	4.7 mm	5.3 mm	5.7 mm	0 mm

**Table 4:** The relationship between the average values of the IRS parameters obtained in the coronal view and the minimal recommended IRS diameters for the second maxillary molars according to the IRS shape (critical points are colored). The last column represents the value of vertical diameter required to achieve implant stability (above 10 mm).

The Interradicular Septum Shape	Level A	Level B	Level C	Level D	10 − (h + H)
Arrow	2.5 mm	3.5 mm	4.4 mm	5.5 mm	2 mm
Boat	3 mm	3.5 mm	4 mm	5.4 mm	2.5 mm
Drop	2.3 mm	3.3 mm	3.3 mm	3.8 mm	1.2 mm
Palatal convergence	2.7 mm	3.7 mm	4.6 mm	5.2 mm	1.8 mm
Buccal convergence	2.3 mm	3.3 mm	4 mm	4.4 mm	0.5 mm

**Table 5:** The relationship between the average values of the IRS parameters obtained in the axial view and the implant surface area for the first maxillary molars according to the IRS shape (critical points are colored).

The Interradicular Septum Shape	Arrow	Boat	Drop	Palatal Convergence	Buccal Convergence
Surface area at level A	5.7	4.7	3.7	6	4.8
Surface area at level B	8	5.1	7.4	9.7	7.3
Surface area at level C	9.3	5.6	7.8	13	10
Surface area at level D	12.4	8.5	6.4	13.2	9.7

**Table 6:** The relationship between the average values of the IRS parameters obtained in the axial view and the implant surface area for the second maxillary molars according to the IRS shape (critical points are colored).

The Interradicular Septum Shape	Arrow	Boat	Drop	Palatal Convergence	Buccal Convergence
Surface area at level A	3	3.7	2.2	3.3	2.1
Surface area at level B	4.6	5.2	3.3	5.5	3.1
Surface area at level C	5.8	5.4	3.2	6.4	3.5
Surface area at level D	7.2	6.7	3.1	6.7	5.9

## Data Availability

Data available on request from authors.
